# An odyssey in the space of molecules, genes, biology and brain: an interview with Sabine Cordes

**DOI:** 10.1242/dmm.019893

**Published:** 2015-02

## Abstract

Sabine Cordes is currently Senior Investigator at the Lunenfeld-Tanenbaum Research Institute, where she studies the genes involved in craniofacial and neuronal development and psychiatric disorders. Although now renowned as an excellent mouse geneticist and neurobiologist, she is actually a biochemist by training. Indeed, she started her career at the Department of Biochemistry at Berkeley, University of California, studying ethylene-induced gene expression during tomato fruit ripening with Robert L. Fischer. She then became fascinated by brain development and decided to join Greg Barsh’s lab at Stanford University to work specifically on hindbrain segmentation. Her interest in psychiatric disorders was, in her own words, ‘accidental’. In this interview, Sabine recounts the interesting steps that took her from the study of chemistry and molecules to that of genes and mouse genetics, to researching on neurodevelopment and mood disorders. She also shares with us her personal forward-looking view of biomedical science, based on her own experience and on the impact of new advances that are revolutionising our understanding of cell biology and neurobiology.

Sabine Cordes obtained her undergraduate degree in Chemistry from Berkeley, University of California, where she also got her PhD from the Department of Biochemistry. After this training in Chemistry and Biochemistry, she moved into a postdoc position at Stanford University, where she joined Greg Barsh’s lab to study genetics and neurodevelopment. In that period, by using mouse genetics, she investigated the role of *Kreisler* (now known as *MafB*), a gene encoding a basic domain leucine zipper transcription factor, in hindbrain segmentation in vertebrates. After spending almost 7 years at Stanford, she moved to the Lunenfeld-Tanenbaum Research Institute at Mount Sinai Hospital in Toronto, where she obtained grants from the Medical Research Council of Canada and Canadian Institute of Health Research to continue studying hindbrain development. In addition, she also obtained a grant from the Edith and John Low-Beer (EJLB) foundation that allowed her to branch out to study later stages in cranial nerve development and the role of the serotonergic system in mood disorders. Her team has recently discovered a link between the gene *gumby* (also called *Otulin*) – which encodes a deubiquitinase – and angiogenesis during craniofacial and neural development. Sabine is now Senior Investigator at the Lunenfeld-Tanenbaum Research Institute and has also recently joined the team of academic editors at *Disease Models & Mechanisms*.

**Figure:**
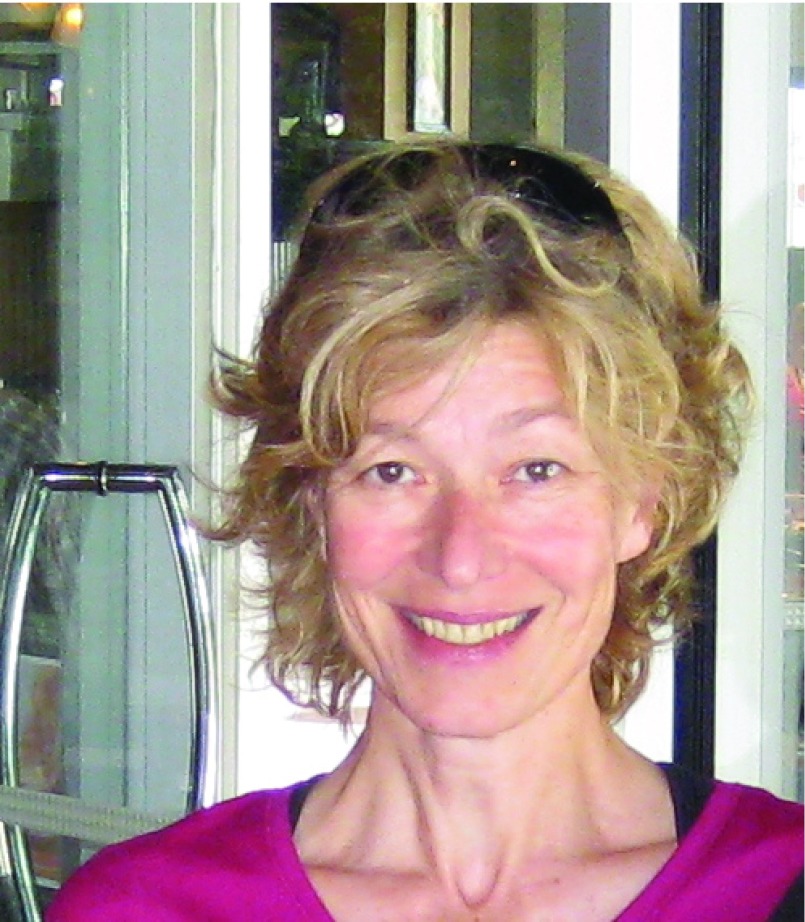


**While training, you did a lot of chemistry. Why did you decide to change to developmental biology and genetics?**

What I found frustrating in biochemistry is that I’d be working on molecules and genes and I wouldn’t know whether they were actually relevant to an organism. Instead, I wanted to know: am I just studying an interaction, am I just seeing what protein binds to DNA, what is it actually doing in a cell and in the organism? That’s why, for my postdoc, I really wanted to do genetics and, in particular, developmental genetics in the mouse.

**But why development in particular?**

I just thought it was beautiful and fascinating, and I cannot give you a more ‘intellectual’ answer. It was not a big plan: it was more that I really loved it. I really loved reading and thinking about development and that’s what I wanted to do. So as a postdoc I joined Greg Barsh’s lab and I was actually an outlier in that lab. Greg Barsh was a great mentor. He is a mouse geneticist and the majority of his lab was studying coat colour. I thought coat colour was interesting but it didn’t really excite me. So when he told me about another project on the classical *kreisler* hindbrain patterning mutants, I decided to join the lab to work specifically on that. *kreisler* was this odd mouse mutant [bearing mutations in the *Kreisler* (*MafB*) gene] that Paula Hertwig had identified in 1939 and Martin Deol described in 1964, and it looked like it was associated with neural tube and segmentation defects. At that point hindbrain segmentation had just come to the forefront because the segmentation of the Hox genes had just been discovered within the rhombomeres. I just thought that the *kreisler* mouse mutant was gorgeous. So that’s why I joined that lab and my overall goal was to further investigate this mutant and the role of the *Kreisler* gene in hindbrain segmentation. One of the most gratifying things is that I was hoping that I would find something that was unique to vertebrates, because it’s so much easier to do genetic screens in flies and worms and other organisms, so why suffer so much with mice? It was really gratifying to discover that *Kreisler*, which is now referred to as *Mafb*, had a role that was entirely unique to vertebrate segmentation of the hindbrain.

Greg was an ideal mentor for me because he was incredibly good at guiding me, but was also kind enough to give me the space to do what I had to do. Plus, the environment at Stanford was terrific! The Developmental Biology programme had just started and they had attracted a bunch of incredible researchers to work all in the same building, so that was an incredibly dynamic time. Stanford had a collegiate and inclusive atmosphere. We had a joint group meeting with Roel Nusse’s group, who was working on Wnt signalling; we were next door to Irv Weissman, who had a very large lab at that time and was also starting a company. Jerry Crabtree’s lab and Ute Francke, who was working on human genetics, were around the corner. Upstairs, there were fantastic developmental biologists such as Matt Scott, Minx Fuller and David Kingsley. So the environment was superb. That’s why I tell students, if you are looking for a postdoc try to find the right dynamic environment.

“Because I started as a biochemist and molecular biologist and then became a geneticist, I really appreciate the importance of a dynamic environment and of collaborations in science”

**How many years did you spend at Stanford? And how did the move to the Lunenfeld-Tanenbaum Research Institute in Toronto come about?**

I spent almost 7 years at Stanford. Mouse genetics research is slow, and I also spent a couple of years looking for a job and trying to choose the one that suited me best. The job offer I liked the most was the one from the Lunenfeld-Tanenbaum Research Institute [LTRI; at the time known as the Samuel Lunenfeld Research Institute]. This is actually a small research institute and doesn’t have tenure positions. But what’s beautiful about it is that it has amazing colleagues. When I went to the interview, and this is still true today, I felt that I could relate to, and talk science with, every single colleague of mine. That really stood out. I had offers from other interesting places and I liked them too but I thought that the LTRI was unique in its diversity. Because I started as a biochemist and molecular biologist and then became a geneticist, I really appreciate the importance of a dynamic environment and of collaborations in science. The LTRI is across the street from the University of Toronto, where I and many of my colleagues have cross-appointments in the Department of Molecular Genetics, and which provides me with my most valuable collaborators: my talented graduate students. This opens up many further avenues for discussions and collaborations. Another attractive feature is that the primary focus for P.I.s at the LTRI is research and, while I do some teaching, I still have some time to do experiments myself. All the work in my lab starts out with a mouse mutant, because I want to know whether or not a gene matters. But then we have to go pretty deep molecularly into mechanisms. So this kind of research really benefits from the environment that I am in and the surrounding facilities. Here in Toronto I have great colleagues within the LTRI, at the University of Toronto and at the surrounding other research institutes, including neurobiologists like John Roder, Joe Culotti, Mei Zhen, Kenichi Okamoto, Sheena Josselyn and Paul Frankland, the mass spectrometry gurus Anne-Claude Gingras and Brian Raught, the exceptional structural biologist Frank Sicheri, RNA expert Ben Blencowe, metabolomics expert Jim Dennis, fly geneticist Howard Lipshitz and iPSC specialist James Ellis, just to name a very, very few! Each one with a different approach and a different way of looking at things and all of them very generous in sharing their knowledge with myself and my students.

“All the work in my lab starts out with a mouse mutant, because I want to know whether or not a gene matters. But then we have to go pretty deep molecularly into mechanisms. So this kind of research really benefits from the environment that I am in and the surrounding facilities”

**And it’s in Toronto that you got interested in psychiatric disorders? Could you tell us how and why?**

It’s actually very funny, because I was completely focused on the hindbrain and on early hindbrain patterning and segmentation. But one day Alan Bernstein, who was the Director of the Institute when I started, walked into my lab and said: “Sabine, you work on the brain?” and I said “I work on the hindbrain”; he said “You work on the brain, you work on schizophrenia!”. I was kind of irritated at the beginning because that didn’t describe what I worked on. But then he explained the reason he wanted me to think about that. He said, “There is a unique foundation called EJLB [Edith and John Low-Beer] and it funds both basic and clinical research that is relevant to schizophrenia. So why don’t you think about that?”. So I thought about it for a while and I ‘discovered’ that serotonergic neurons are born just a short while after the time period I was interested in in early hindbrain patterning, which in the mouse occurs between 8 and 9 embryonic days. So the serotonergic neurons start to appear around 9.5 days and then, between 10.5 and 14.5 days, the majority of these neurons are born. Upon reading more I realised how important the serotonergic system was. Then I started to think about how to design screens for serotonergic mutants and thought that perhaps some of our hindbrain patterning mutants or mutants that affect early development of specific cranial nerves might also affect serotonergic neuron development and so impact mood disorders. Then, of course, it’s not too far of a stretch to start to realise that a lot of craniofacial syndromes are accompanied by behavioural anomalies. So I applied for this grant and the EJLB funded it. I’ll tell you more about this because I think this is important for the readers and the public.

The EJLB was funded by Edith and John Low-Beer, who were wealthy industrialists from Montreal. They had three children, two girls and a boy. Sadly, their son developed schizophrenia and committed suicide. So they decided to establish a foundation – they realised that a lot was unknown about schizophrenia and they wanted to fund research. They did a very admirable job. They set up a scientific board, members of which included Tom Jessell and other outstanding neurobiologists, to select six junior faculty members as scholars every year whom they funded for 3 years for CAN$100,000 per year, which is a lot. When I proposed our screens for serotonergic mutants and their analyses and proposed also the craniofacial work from which *gumby* came, they funded that. I will tell you that the national foundations would not touch that – they rejected that grant with the overarching comment that it was too risky.

“When I proposed our screens for serotonergic mutants and their analyses and proposed also the craniofacial work from which *gumby* came, [the EJLB] funded that. I will tell you that the national foundations would not touch that – they rejected that grant with the overarching comment that it was too risky”

I met Edith when she was in her 90s – she was a remarkable person, who was deeply engaged in fostering scientific understanding of mental health disorders and social solutions for those suffering from mental health challenges. The great part of this private endeavour was that it funded both basic and clinically relevant research and things were really set up right, by having a scientific review board and a rigorous but scientifically open-minded selection process. However, the weakness of this was that, when Edith died, so did the support and enthusiasm for funding science, and now the EJLB Foundation no longer funds scientific research. So that shows both the advantages of that type of endeavour and also shows where it fails. But, having said that, these individual efforts can make a huge difference. And they certainly made a huge difference for my science.

**What have we learnt so far about the role of serotonin in schizophrenia and mood disorders, and what is it that we still need to understand?**

I think in the last 10 years things have changed a lot. Ten years ago the hope was that significant inroads would be made to understand the genetics of depression via large-scale GWAS [genome-wide association studies]. It turned out that, while a few hits came out from the search for bipolar disorder genes, such as *Ankyrin3* and some of the calcium channels as well, depression has been remarkably resistant to offering up good hits. Some of the best associations have actually been serotonergic; for example, TPH2 [tryptophan 5-hydroxylase 2] mutations have been associated with major unipolar depressive disorder. All of that pointed to several things. One is that there are probably many factors that contribute to neuronal health and vitality whose dysfunction might lead to neuronal impairment in these disorders. A number of unpublished works support the idea that there are a lot of modifiers that come into play to generate a healthy serotonergic system, and so you would have many small effects leading to an individual being more resistant or vulnerable to depression. Perhaps from the outset the overall lack of objective measures for diagnosing depressive disorders and allowing us to subcategorize them further may be one of the biggest hurdles: one of the biggest challenges for depression is that, in the clinic, people with depression are self-diagnosed subjectively by interview and there aren’t any additional measures that are non-subjective and indicate reliably what type of disorder you have. Then another thing that I think will emerge is that we’ll start thinking of depression as possibly sometimes having a physiological rather than necessarily neurobiological contribution. So, when I take my pup to the vet, the vet will ask me whether or not ‘Fluffy’ (not my pet’s name) is feeling ‘depressed’. The vet cares about my pet’s mood as an indicator of its energy level, vitality and motivation, ability to take joy in ordinary activities, such as catching a ball, and as an overall indicator of its physiological health. If I say “Fluffy is droopy”, my vet will check Fluffy out for anything that might be physiologically wrong. So that’s the thing that is popping up. Depression is such a complex disorder and may fall into perhaps four (or more) major categories: one is that you’ll have true neurobiological depression that really is a neurobiology disorder; the others are that you have something linked with, either caused by or co-occurring with, inflammatory, cardiovascular or metabolic disorders.

With respect to research approaches, a shift that I’m seeing in our work in the lab, and that of others, is that, while the focus before has been heavily on gene identification, we are beginning to turn our efforts to understanding the cell biological roles of these genes and really towards understanding the cell biology of the neuron. In some instances, the roles really are ‘neuron-specific’, but in other cases neurons, given their long lives and specialised processes, may just be more sensitive indicators – the proverbial ‘canary in the coal mine’ – for cell biological processes malfunctioning or not functioning optimally.

“…a shift that I’m seeing in our work in the lab, and that of others, is that, while the focus before has been heavily on gene identification, we are beginning to turn our efforts to understanding the cell biological roles of these genes and really towards understanding the cell biology of the neuron”

One of the hopes in the field is that combining results from genetic analyses in people and model organisms with increased understanding of the cross-relatedness of physiological and neurobiological health (perhaps also on the cell biological level) may help us meet the challenge of coming up with accurate indicators or biomarkers. For the sake of accessibility, blood-based assays may be the most useful; for example, analysing patients’ blood before they have been treated with antidepressants, during and after, to see what the markers are actually telling us and to predict response to therapy. For depression, cognitive behavioural therapy used in conjunction with drugs targeting the serotonergic system is still one of the most successful treatments. But about 40% of the population doesn’t respond to serotonergic drugs and we need objective indicators to understand why it is so.

**So you mean trying to get the whole picture of an individual, and combining cognitive and behavioural tests with biomarkers?**

Yes, and you want to use both the very basic as well as clinical knowledge, because we’ll never know where the breakthroughs that will allow us to really make advances will come from. And I want to remind the readership that the reason why people became interested in serotonin and the serotonergic system was an accident – people had discovered monoamine oxidases and thought that monoamine oxidase inhibitors would make a good treatment for tuberculosis. What they found was that people in the clinical trials being treated with those inhibitors against tuberculosis were really happy compared to controls. And that was entirely fluky. It’s not something that we would have predicted. That’s why you want to interest a broad range of scientists to work on a problem. Right now, the pressure in Canada is that really every research grant is supposed to have considerable ‘translational’ relevance and ideally ‘translational’ applications, and I think that’s a dangerous pressure because it assumes that I know what’s going to be useful 20 years down the road and can accurately predict negative side effects. But if I already presumptively know all of that, why should I or anyone else bother doing research at all? So there’s a real danger there. Basic research and multidisciplinary environments are very important. Again, what I really enjoy about where we are is that our institute sits right next to the University of Toronto campus, and it’s right next to a bunch of research institutes, such as the CAMH – the Centre for Addiction and Mental Health – and the Hospital of Sick Children, so we have also clinician scientists, such as Albert Wong, as collaborators. The cross-talk with clinicians is really helpful to us. At the same time we are surrounded by people using complementary structural biological and proteomics strategies, and microscopy experts, such as the optogeneticist Kenichi Okamoto, whom I’ve already mentioned, and electrophysiology-imaging whizz John Georgiou, down the hall from us. So, for each problem we are facing we decide whether to focus on a cell biological imaging, proteomic, structural or genetic approach, and often we end up combining our forces and using all of these strategies to fully understand any given problem.

Another thing that’s been really interesting is that, when I started working on schizophrenia, I was deeply embedded in the neurodevelopmental hypothesis for mood disorders. Certainly there can still be a neurodevelopmental component but, surprisingly to me – maybe the rest of the field might know more – while we have been working on mouse mutants and others have been studying ALS, Parkinson’s and other disorders, in parallel we’ve been migrating towards RNA and ubiquitin-based mechanisms and cell biological mechanisms. This was not by design. It is just entirely where genetics and mouse genetics have been taking us. If you had asked me 10 years ago, I wouldn’t have said “Oh yes, let’s work on this”. Twenty years ago people would have said “Let’s all work on neurotransmitter receptors”, 10 years ago “Let’s study the axon guidance and early synaptogenesis molecules”. All those molecules are important but, surprisingly, it’s the cell biological processes that are governed by ubiquitination, by RNA and its splicing and mobility, that more recently have been emerging as being absolutely central and possibly more global and affecting not just a single molecular interactions but entire programmes of axon guidance molecules, neurotransmitters and protein interactions.

**So you said what our view was 20 and 10 years ago. I am sorry but I can’t help asking: what do you think we have ahead of us in 10 years’ time?**

Well, I proved that I can’t predict such things. The only thing that I have any confidence in is that, if we use genetics and very objective scientific measures, and use the techniques that are available and their advances, we will keep discovering incredibly interesting things. I think that right now, the major advances that have been fuelling things are the ability to use CRISPR, the enhanced proteomic methods that allows us to zoom in on possible functions and interactions within large gene networks and then look into proteomic interactions and the interactomics, so that genetic and protein interactions start to make sense. And then I think also the increased cell biological techniques and knowledge. Probably, in 10 years we’ll realise we had an overly simplified view of what’s actually going on in the cell. So, as our ability to dissect that evolves, I think we’ll be humbled by how beautiful this is. It will become clearer and we’ll begin to appreciate, for instance, the diversity of RNA particles and what they are actually doing, where they are going, and how they are regulated. Right now our vocabulary that describes these things is limited. I teach a class on molecular mechanisms in neurobiology and psychiatric diseases and our vocabulary that describes what’s going on sub-cellularly is crude at best. I think it’s going to be pretty funny looking back 10 years from now and seeing what we thought was going to happen. If I only apply that to me, 10 years ago I would not have thought that RNA- and ubiquitin-based mechanisms would be what I was studying and really throwing myself into. Certainly not ubiquitin. I would have said, “This is just something yeast people talk about forever and I don’t care about. It’s probably just a marker of neurodegeneration, as in Parkinson’s disease; it doesn’t have anything to do with the real primary thing that’s happening”.

**But after *gumby* you changed your mind?**

Yes, I did. And the fact that *gumby* and linear (de)ubiquitination doesn’t occur in all animals is gratifying. Again, as I told you, I was really happy when *Kreisler* turned out not to be a standard segmentation gene also in flies and worms. I want to stress that the comparative and evolutionary conservation is really important and highlights ancient, essential mechanisms. Obviously, it’s really important that people study those. But I think it is important and fascinating to understand why we are mammals, why we have a backbone, what’s so different about us. It’s been very surprising and gratifying that *gumby* ended up emerging in urochordates and chordates and that its emergence coincides with more complex intracellular communication and unique organismal interactions with their environments. We have focused on *gumby* developmentally and continue to do so. However, the functions of linear ubiquitination that were identified first are its modulation of inflammation and adaptive immunity. If you think about the environmental challenges that we face, who is trying to attack us versus which organism is trying to attack a fly, these are quite diverse and different. From an evolutionary perspective it is enjoyable to see how different organisms deal with challenges in life. So certainly *gumby* and linear (de)ubiquitination are contributing to our understanding of how that happens.

**How do you relax outside the lab?**

I like to spend my time outdoors, running. We actually live close to a lake so we can paddle in the summer, surprisingly. I know people don’t think of Canada as warm, but in summer it is. I like keeping active, being with friends, reading, walking the dogs on the beach. The other thing I do, or maybe I’m doing the same thing just in a different area, I love planning adventures. So every year I plan ‘candidate’ adventures and I put them in front of my husband as grant applications. We usually try to pick one that really makes a difference: one example, we went to the Galapagos with some close friends but we also spent some time with Earth Watch and the Cheetah Conservation Fund. And the idea there is that I feel that molecular genetics and genetic research might not just be valuable for human disease but can make a huge impact in helping to preserve species. For example, Laurie Marker and her team at the Cheetah Conservation Fund have started to use this technology to try to track this very elusive, often solitary, species and figure out where the individuals are going. I think that it’s our responsibility to look out for those that cannot speak for themselves, so in this case wild animals, and to try to do some good when we are travelling and outside of work.

